# Simultaneous cancer control and diagnosis with magnetic nanohybrid materials

**DOI:** 10.3762/bjnano.7.14

**Published:** 2016-01-27

**Authors:** Reza Saadat, Franz Renz

**Affiliations:** 1 Institute for Inorganic Chemistry, Leibniz University Hannover, Callinstr. 3–9, 30169 Hannover, Germany

**Keywords:** biocompatible nanoparticles, cancer control, cancer diagnosis, magnetite nanoparticles, positron emission tomography

## Abstract

Coated magnetite nanoparticles were linked to ^68^Ga complexes used in the positron emission tomography (PET) for a new technical approach to detect cancer tissue with radiopharmaceuticals. By substitution of the Ga isotope with an alpha emitter the same compound could be used for cancer treatment. Furthermore the nanoparticles were connected to pH-sensitive complexes, enabling a pH-controlled assembly/disassembly and therefore the spreading of the particles in the tissue. With this novel method of combining detection and treatment simultaneously, the amount of medical exposure could be minimized for the patient. The results demonstrate that magnetite nanoparticles can effectively be functionalized with PET isotopes and pH sensitive complexes in order to use them as a new type of radiopharmaceuticals.

## Introduction

**Figure 1:**
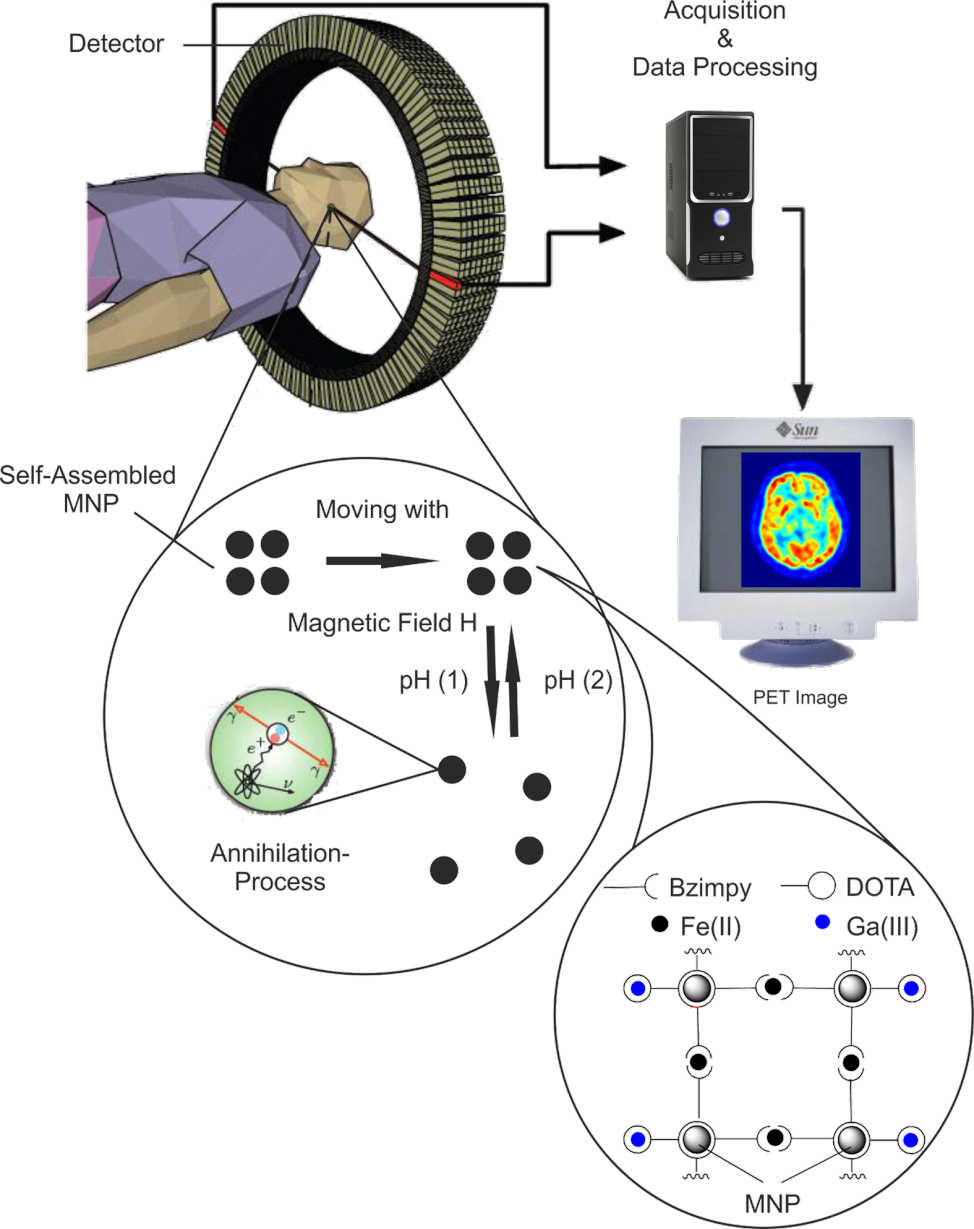
Model of a nanohybrid-PET-system. MNP can assemble and disassemble at different pH. The disassembled NP spread in the tumor cell and can be detected by PET [6–7].

In the field of diagnostic investigation of metabolic diseases imaging methods based on nanotechnology have been recently gaining more and more importance [1–2]. In the last 30 years the positron emission tomography (PET) has acquired relevance among the current conventional imaging methods such as X-ray, computed tomography (CT) or magnetic resonance imaging (MRI) [3]. PET gives insights in the metabolism of living organisms and allows to detect diseases of neurological or oncological origin [4–5]. It is a nuclear medicine imaging technique that produces 3D images of functional processes in the body. The circularly arranged system detects high energetic γ-quanta, emitted indirectly via annihilation of a positron with an electron by a radiopharmaceutical called “tracer” which has been previously injected intravenously into the patient. A computer reconstructs then tomographic images of the tracer concentration so that sectional images can be conveyed [5]. By linking PET isotopes to magnetic nanoparticles (MNP) a sophisticated system with different applications can be achieved (Figure 1): The magnetism of the particles allows an all-time control of the nanopharmaceuticals position, a linker, connecting the nanoparticles to each other, enables a pH-controlled assembly/disassembly and the PET-isotopes provide a detectable annihilation signal.

There are several isotopes that can be used in the PET: Besides the mostly used isotopes namely ^18^F, ^11^C, ^13^N and ^15^O, ^68^Ga is often used since it can be easily extracted by a ^68^Ge/^68^Ga generator [5–6,8–9]. The ^68^Ga isotope is complexed for example by a phosphonate containing chelate ligand and injected into the body. The tracer enriches in the hydroxyapatite (Ca_10_(PO_4_)_6_(OH)_2_) and can be used for investigations of the bone metabolism [10–12]. Modifications of the functional groups of the ligand enables the use of the Ga complex in a different way: PET-analogue Ga-DOTA (DOTA = 1,4,7,10-tetraazacyclododecane-1,4,7,10-tetraacetic acid) complexes were synthesized and linked to Fe_3_O_4_ MNP. In this manner it is possible to transport the PET tracer with an external magnetic field right to the target location. The functionalized MNP are linked to each other via [Fe(II)(bzimpy)_2_] (bzimpy = 2,6-bis(benzimidazol-2-yl)pyridine). The resulting MNP cluster can be used as a pH-labile switch: By protonation of the electron pairs of the ligands (which are coordinated to the iron ion) the MNP cluster-stabilizing complex [Fe(II)(bzimpy)_2_] decomposes [13] in an acidic environment such as the one present in the cancer cell (Figure 1) [7,12]. Equipped with an alpha emitter [14], they could initiate an apoptosis of the tumor cells. This procedure may allow for combining diagnosis and therapy for cancer diseases.

## Results and Discussion

**Figure 2:**
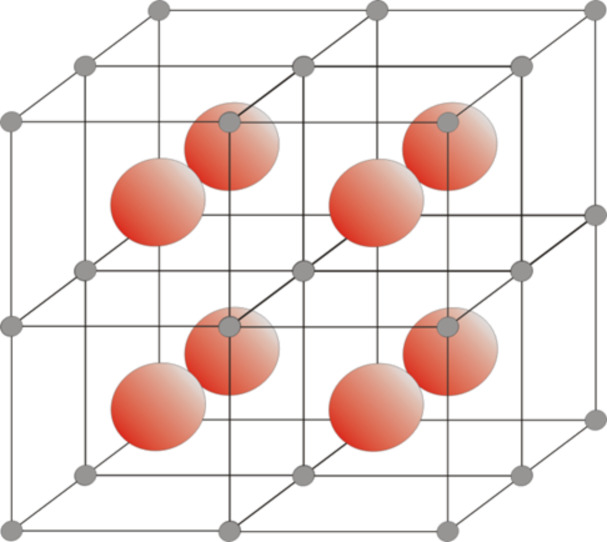
Idealized 3D cave structure of a NP cluster. Grey spheres: MNP, lines: [Fe(II)(bzimpy)_2_] linking units, orange spheres: possible intercalated molecules.

We synthesized magnetite nanoparticles through the well-established co-precipitation method [15] and proceeded with further functionalization to obtain biocompatible particles that can assemble and disassemble by entering media with different pH values. The resulting cave structure consists of three nanoparticle units in every spatial direction. The accruing free space inside the cave structure could be used to intercalate desirable pharmaceuticals (Figure 2).

**Figure 3:**
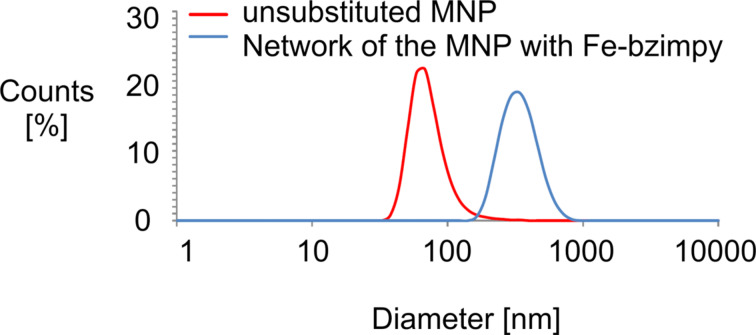
Diameter of the unsubstituted and networked MNP.

To reach a mutual conjunction we used silanized and amino-functionalized MNP (**1**), which were dispersed for 4 h in an ultrasonic bath at 80 °C in acetonitrile with Cl-bzimpy. The deposit was then added to an iron(II) chloride tetrahydrate solution and stirred for 2 h at 25 °C. DLS measurements of the connected MNP (Figure 3 and Table 1) showed a strong increase of the hydrodynamic diameter of the [Fe(II)(bzimpy)_2_]-MNP (**2**) in comparison to the unsubstituted MNP (+275 nm). It can hence be assumed that a network (consisting of about three units in all spatial directions) of the MNP was obtained. We assume that bigger cluster units do not appear long enough to be detected due to shear forces destroying them.

**Table 1:** Comparison of the hydrodynamic diameters.

compound	hydrodynamic diameter [nm]

amine-MNP (**1**)	74.5
Fe(II)-(Cl-bzimpy)_2_-MNP (**2**)	349.8
Cl-bzimpy-MNP (**3**)	123.0
Ga-DOTA-MNP	141.2

The cluster units are stable in a pH range from 13 to 4.5. At lower pH the clusters disassemble as anticipated and exhibit the same hydrodynamic diameter as before. The TG/DTA measurements of Cl-bzimpy-MNP (**3**) showed a weight loss of 2.9% in the range between 30 and 100 °C caused by solvent molecules. In the range from 100 to 500 °C the weight loss was 24.5%, so that it can be assumed that the organic component bzimpy was linked to the MNP (see Table 2).

**Table 2:** Comparison of weight loss during the TG/DTA measurement.

compound	total organic weight loss [%]

amine-MNP (**1**)	4.3
Cl-bzimpy-MNP (**3**)	24.5
Ga-DOTA-MNP	46.1

**Figure 4:**
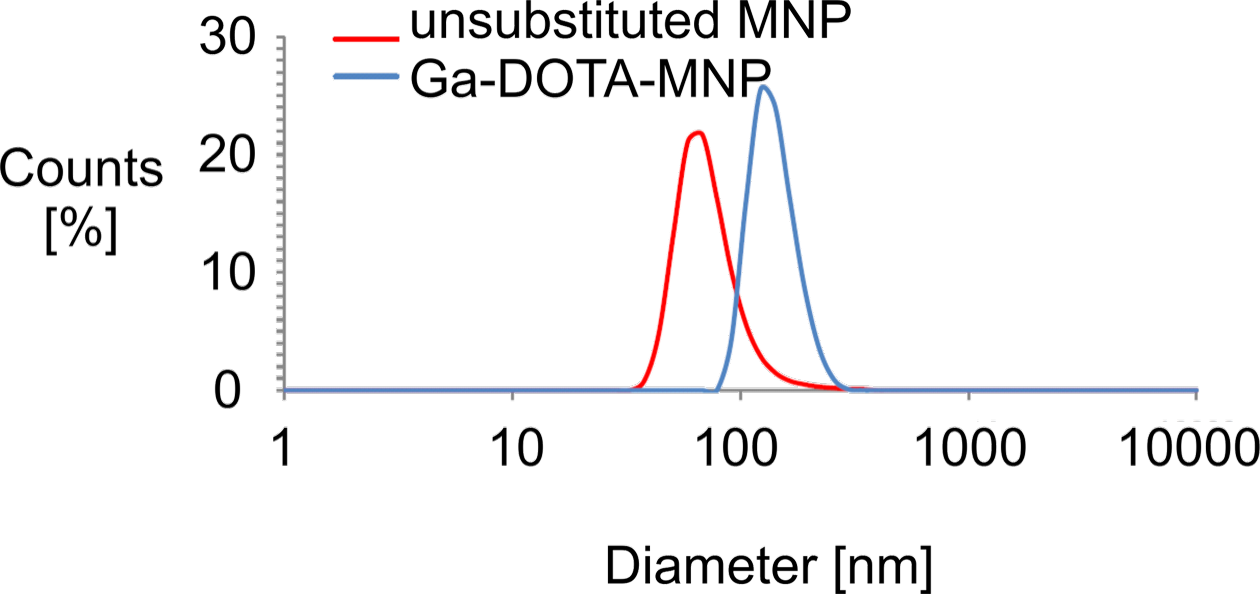
Diameter of the synthesized MNP.

In order to give the yield PET functionality to the MNP we chose the DOTA chelator. It is highly preferred in medical applications caused by the high stability of various metal ion–DOTA complexes [16]. In this study we used a non-radioactive Ga isotope as a proof of principle due to its same chemical behavior compared to the radioactive isotopes of Ga. By simply using unmodified DOTA and solving it in heated dimethylformamide (DMF) we can use standard coupling reagents for the functionalization of the MNP. By a high amount of solvent the connection of the MNP to each other caused by two carbon acid groups of the DOTA is prevented. The conjunction of the Ga-DOTA complex with the MNP was performed through Steglich esterification [17]. The silanized and amino-functionalized MNP were dispersed with Ga-DOTA, DCC and 4-DMAP in dimethylformamide for 24 h at 80 °C in an ultrasonic bath. DLS measurements showed that the complex was successfully linked to the MNP while the MNP were not linked to each other. An enlargement of the hydrodynamic diameter of ca. 67 nm compared to the unsubstituted MNP was observed (Figure 4 and Table 1).

**Figure 5:**
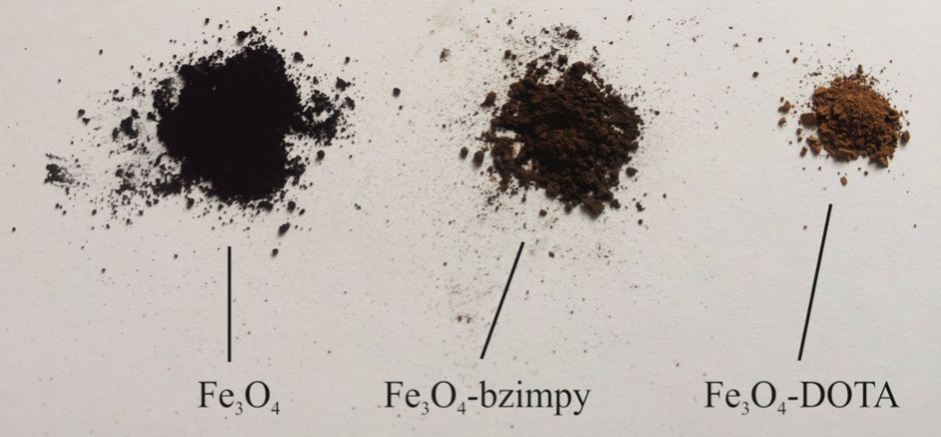
MNP after different functionalisation steps.

TG/DTA measurements detected a weight loss of 9.6% till 100 °C caused by solvent molecules. The weight loss of 46.1% was caused by the burning organic component in the range of 100 to 550 °C. The unsubstituted MNP were also measured and compared, in order to demonstrate, that the Ga-DOTA-MNP had a significant higher organic component (Table 2). Noticeable is the fact that with increased functionalisation the MNP get brighter (Figure 5).

The particles were washed and dried under the same circumstances so that a coloring influence of different solvent molecules can be foreclosed. Thus the visual input of the MNP can give a first cautious estimation of a successful functionalization.

## Conclusion

In conclusion, Ga-DOTA and [Fe(II)(Cl-bzimpy)_2_] complexes were synthesized and linked to magnetic nanoparticles. The linked MNP were analyzed with DLS and TG/DTA and showed a significant enlargement of the hydrodynamic diameter compared to the unsubstituted MNP, which is clearly an indication for a successful conjunction to the Ga-DOTA as well as the [Fe(II)(Cl-bzimpy)_2_] complex. The higher weight loss of the modified MNP detected with TG/DTA confirms a succesful synthesis of the desired compounds (see Supporting Information File 1 for full experimental data). Further synthesis and studies such as the simultaneously affixing of the mentioned complexes on the MNP, the labeling with alpha emitters, the precise pH stability and the behavior of the MNP in a specified magnetic field are planed.

## Supporting Information

File 1 Additional experimental data.
