# Supplementary material for: Simultaneous cancer control and diagnosis with magnetic nanohybrid materials
Source: Beilstein J Nanotechnol. 2016 Jan 27;7:121–5. doi: 10.3762/bjnano.7.14 (PMC4734441; doi:10.3762/bjnano.7.14)
Supplement: File 1 — Additional experimental data. [file Beilstein_J_Nanotechnol-07-121-s001.pdf]

Supporting Information

for

Simultaneous cancer control and diagnosis with magnetic nanohybrid materials

Reza Saadat[*1] and Franz Renz[1]

Address: [1]Institute for Inorganic Chemistry, Leibniz University Hannover, Callinstr. 3–9, 30169 Hannover, Germany

Email: Reza Saadat* - rezasaadat.chem-uni.hannover@mail.de

* Corresponding author

Additional experimental data

Table of contents

1. General information 2

2. Experimental procedures 2

3. Spectra 5

1. General information

All commercially available reagents and solvents were deployed as received from Sigma Adrich Corporation without further purification and without drying. FTIR spectra were performed as KBr pellets with a Bruker Tensor 27 spectrometer. TG-DTA measurements were obtained with a STA 409 from Netzsch with a gas flow of 80% N_2 and 20% O_2. DLS and zeta potential measurements were performed with a Zetasizer 6.01 from Malvern Instruments at pH 7 in water. XRD measurements were obtained with a Stoe StadiP PSD with Cu Kα radiation.

2. Experimental procedures

Synthesis of magnetite nanoparticles

$FeCl_2 \cdot 4H_2O$ (5 g, 25.1 mmol) and $FeCl_3 \cdot 6\ H_2O$ (13.6 g, 50.3 mmol) were solved in 60 mL distilled water. The solution was stirred 20 min after adding 10 mL of an NH_3 solution (25%) at 25 °C. The resulted black deposit was filtered, washed three times with water and dried under vacuum. The yield was 5.46 g.

Silanization of magnetite nanoparticles

5 g of the magnetite nanoparticles were dispersed in 400 mL Ethanol in an ultrasonic bath. Subsequently, 120 mL of an NH_3 solution (25%), 2.33 mL TEOS (10.5 mmol) and 80 mL distilled water was slowly added within 2 h at 25 °C. The resulted black-brown deposit was separated with a hand-held neodymium magnet, washed twice with ethanol and dried under vacuum. The yield was 5.15 g.

Aminofunctionalisation of silanized magnetite nanoparticles

3.5 g of silanized nanoparticles were dispersed with an ultrasonic bath in 500 mL ethanol. Afterwards 15 mL (3-aminopropyle)triethoxysilane (60 mmol) was added and treated with ultrasound for 12 h at 25 °C. The resulted brown deposit was separated with a hand-held neodymium magnet, washed twice with ethanol and dried under vacuum. The yield was 3.37 g.

Conjunction of the aminofunctionalized nanoparticles with Cl-bzimpy

0.03 g of aminofunctionalized nanoparticles were dispersed with an ultrasonic bath in 20 mL ethanol. Subsequently 0.06 g Cl-bzimpy (bzimpy = 2,6-bis(benzimidazol-2-yl)-pyridine, 0.2 mmol) was added and treated with ultrasound for 4 h at 25 °C. The resulted bright-brown deposit was separated with a hand-held neodymium magnet, washed twice with ethanol and dried under vacuum. The yield was 0.029 g.

Conjunction of the nanoparticles to each other

0.02 g of Cl-bzimpy nanoparticles were dispersed with an ultrasonic bath in 40 mL ethanol. 0.27 g $FeCl_2 \cdot 4H_2O$ (1 mmol) and 0.1 g ascorbic acid were added to this dispersion and treated with ultrasound for 2 h at 25 °C. The resulted brown deposit was separated with a hand-held neodymium magnet, washed twice with ethanol and dried under vacuum. The yield was 0.025 g.

Conjunction of Ga-DOTA with the aminofunctionalized nanoparticles

0.15 g of aminofunctionalized nanoparticles were dispersed with an ultrasonic bath in 300 mL DMF. Subsequently, 0.39 g DCC (0.19 mmol), 0.18 g 4-DMAP (1.5 mmol) and 0.1 g Ga-DOTA (0.2 mmol) was added and treated with ultrasound for 24 h at 80 °C. The resulted bright-brown deposit was separated with a hand-held neodymium magnet, washed twice with DMF and dried under vacuum. The yield was 0.152 g.

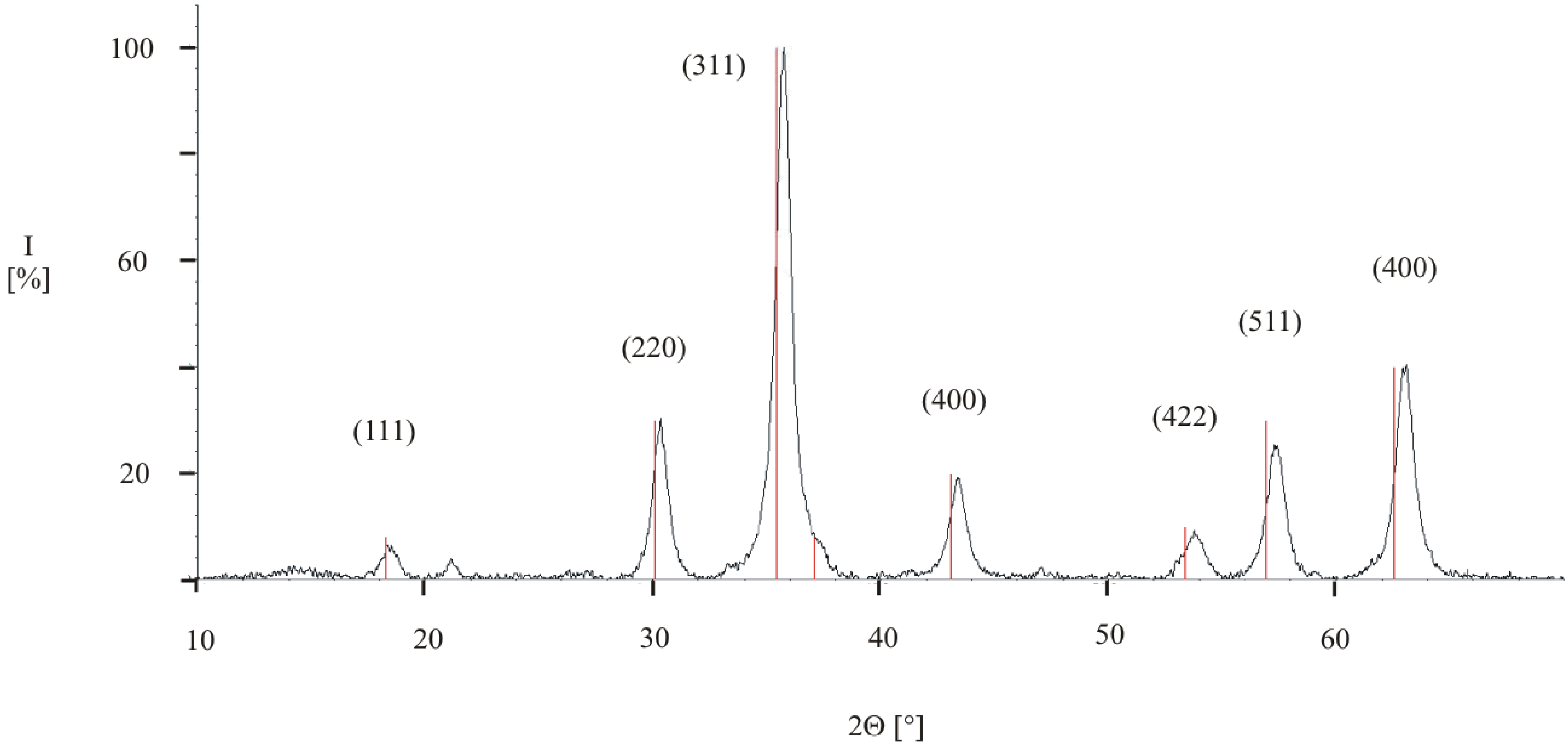

Figure S1: XRD of Fe_3O_4

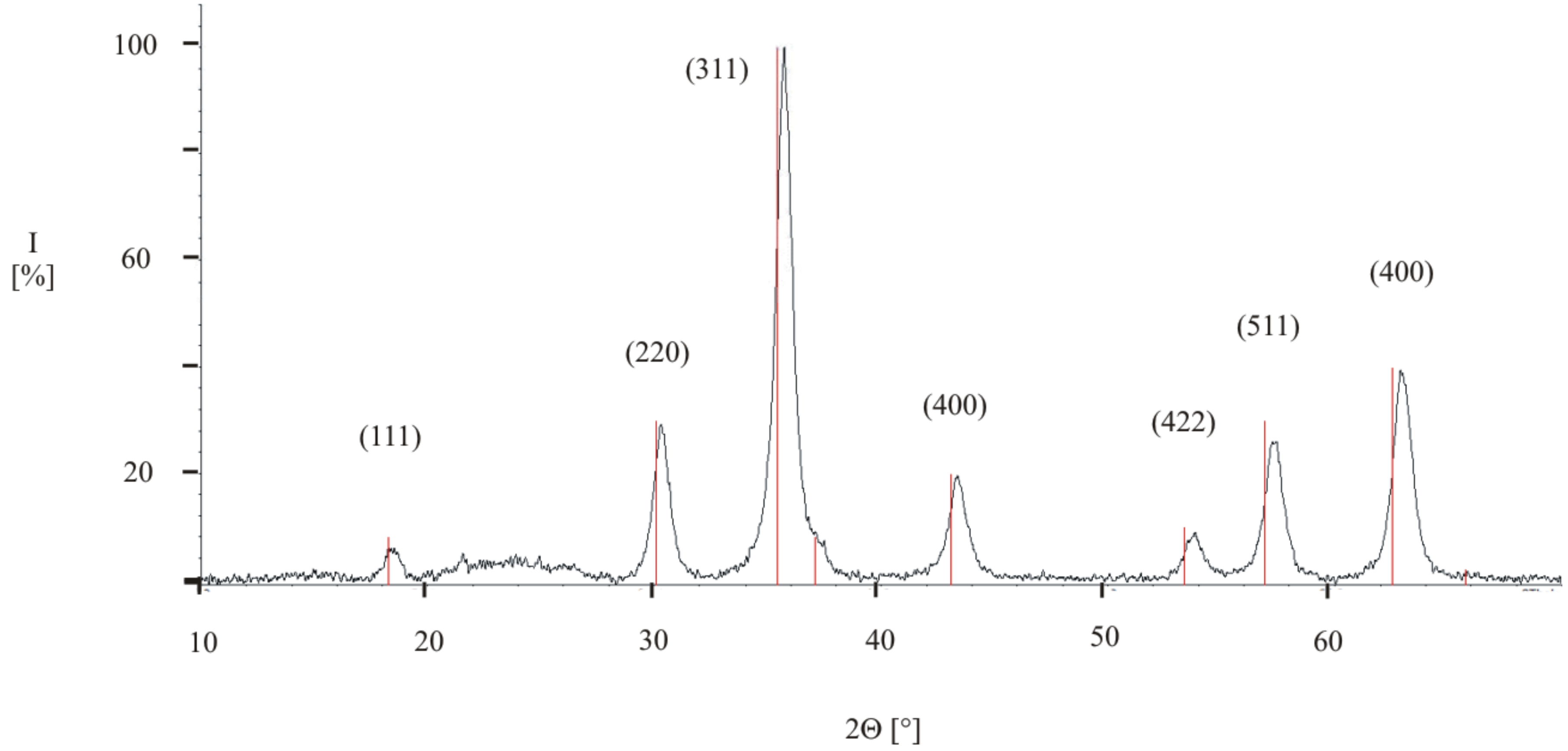

Figure S2: XRD of $Fe_3O_4@SiO_2$

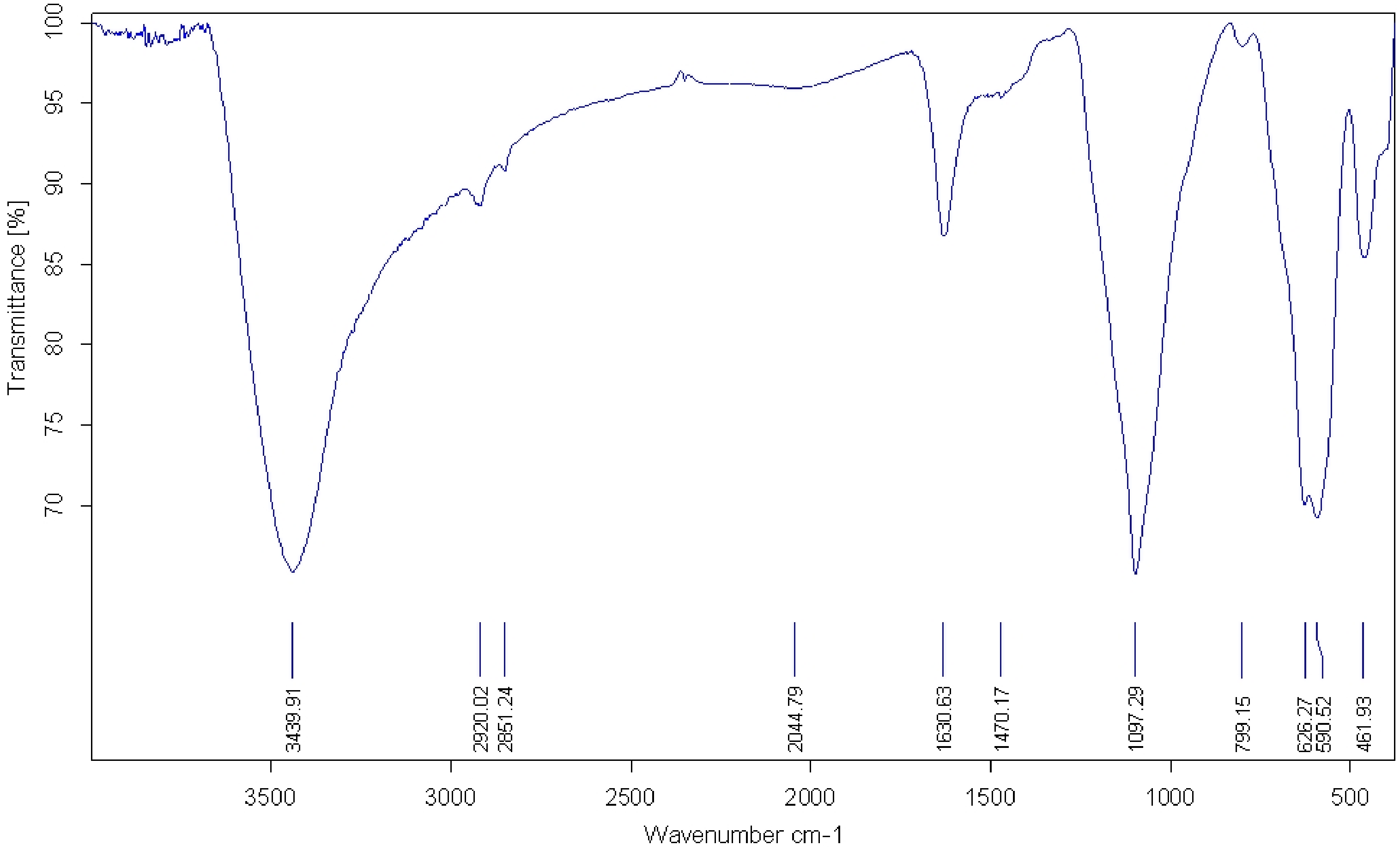

Figure S3: IR spectrum of aminofunctionalized $Fe_3O_4@SiO_2$.

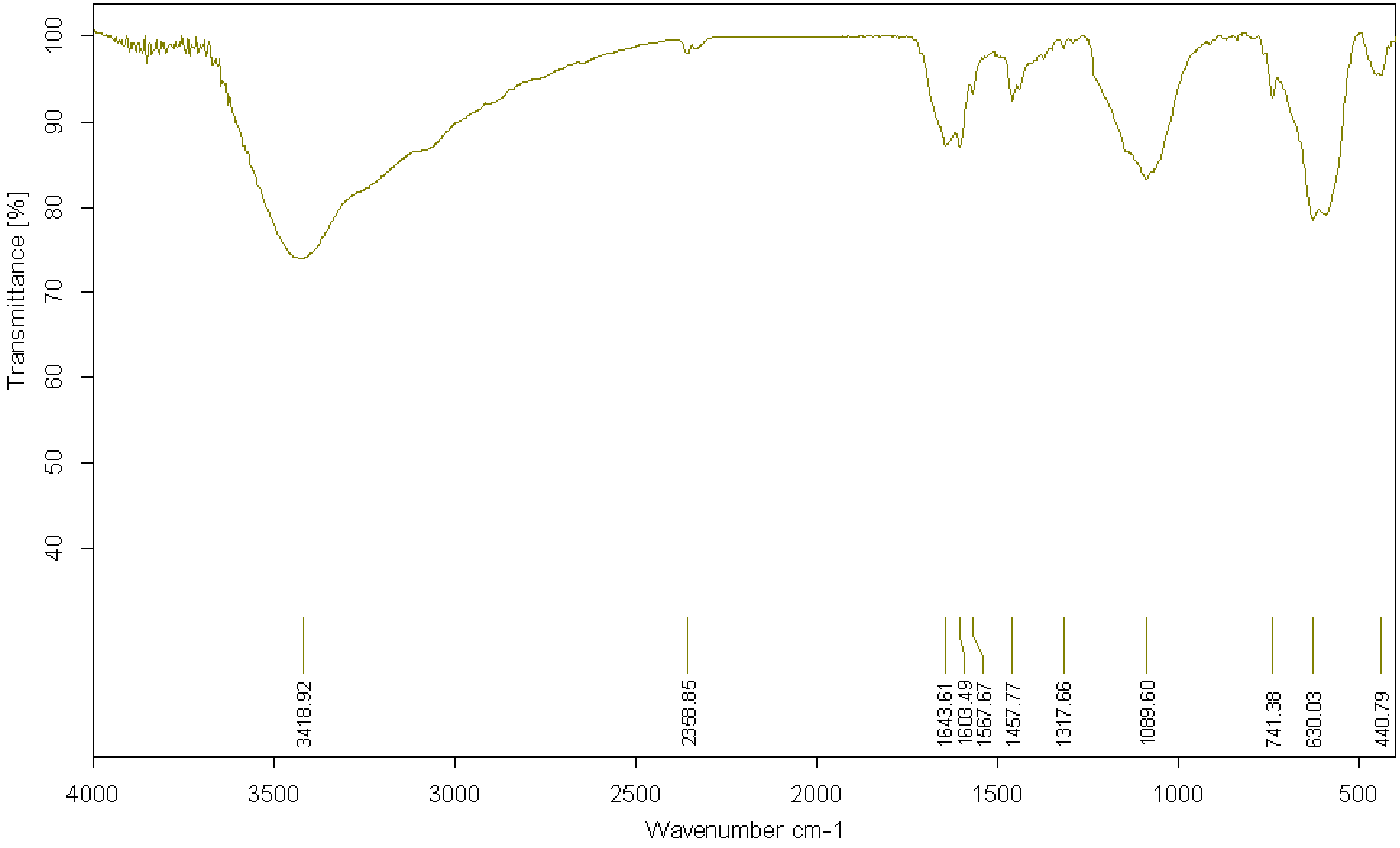

Figure S4: IR spectrum of Fe_3O_4-bzimpy.

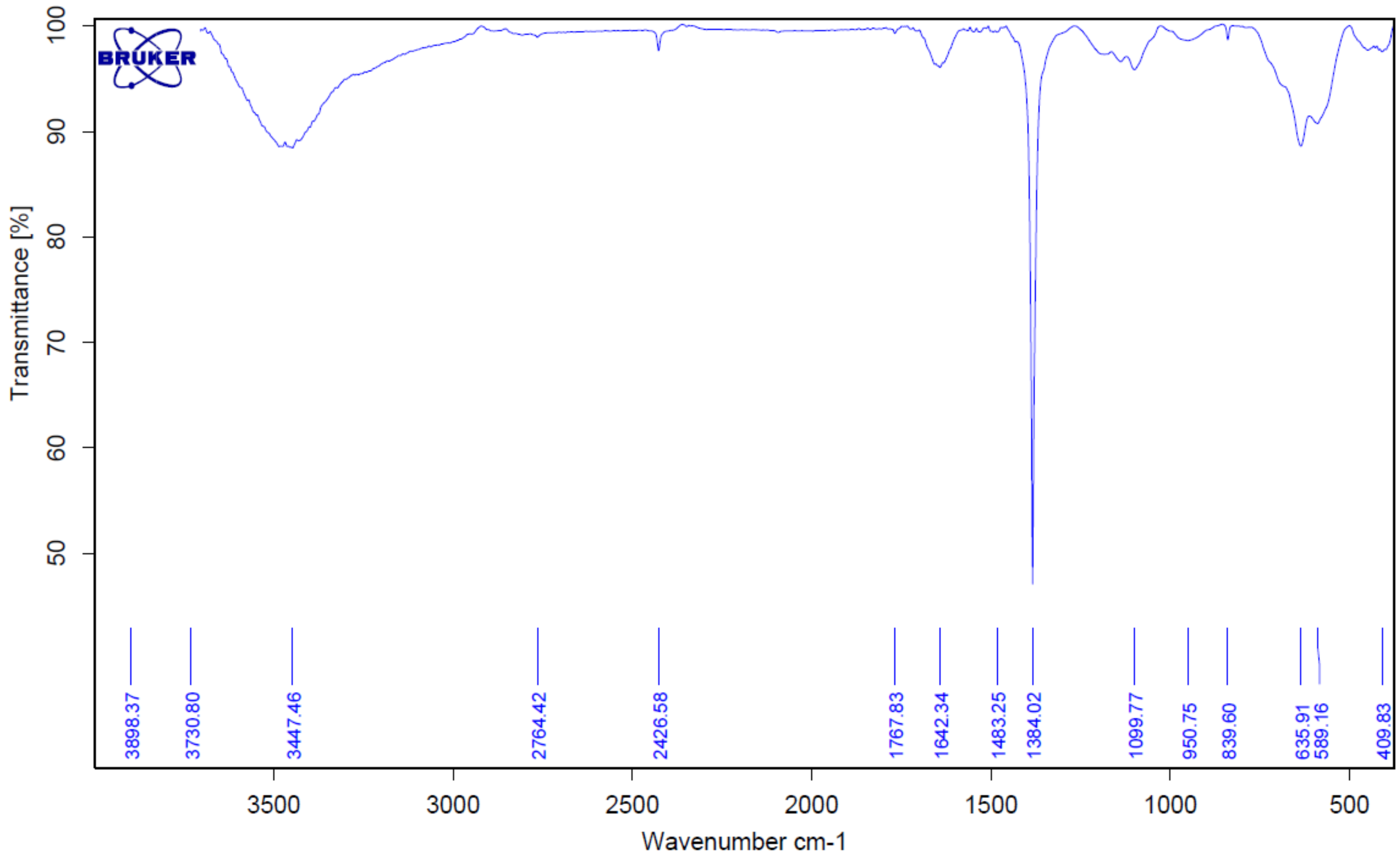

Figure S5: IR spectrum of Fe_3O_4-DOTA
